# ComsystanJ: A collection of Fiji/ImageJ2 plugins for nonlinear and complexity analysis in 1D, 2D and 3D

**DOI:** 10.1371/journal.pone.0292217

**Published:** 2023-10-05

**Authors:** Helmut Ahammer, Martin A. Reiss, Moritz Hackhofer, Ion Andronache, Marko Radulovic, Fabián Labra-Spröhnle, Herbert Franz Jelinek

**Affiliations:** 1 GSRC, Division of Medical Physics and Biophysics, Medical University of Graz, Graz, Austria; 2 Community Coordinated Modeling Center, Greenbelt, Maryland, United States of America; 3 Research Center for Integrated Analysis and Territorial Management, Faculty of Geography, University of Bucharest, Bucharest, Romania; 4 Experimental Oncology, Institute for Oncology and Radiology of Serbia, Belgrade, Serbia; 5 School of Biological Sciences - Te Kura Mātauranga Koiora, Victoria University of Wellington - Te Herenga Waka & Paediatrics Research Unit, Te Whatu Ora | Health New Zealand – Nelson Marlborough, Nelson, New Zealand; 6 Department of Biomedical Engineering and Health Engineering Innovation Center, Khalifa University, Abu Dhabi, United Arab Emirates; 7 Biotechnology Center, Khalifa University, Abu Dhabi, United Arab Emirates; Menoufia University, EGYPT

## Abstract

Complex systems such as the global climate, biological organisms, civilisation, technical or social networks exhibit diverse behaviours at various temporal and spatial scales, often characterized by nonlinearity, feedback loops, and emergence. These systems can be characterized by physical quantities such as entropy, information, chaoticity or fractality rather than classical quantities such as time, velocity, energy or temperature. The drawback of these complexity quantities is that their definitions are not always mathematically exact and computational algorithms provide estimates rather than exact values. Typically, evaluations can be cumbersome, necessitating specialized tools. We are therefore introducing ComsystanJ, a novel and user-friendly software suite, providing a comprehensive set of plugins for complex systems analysis, without the need for prior programming knowledge. It is platform independent, end-user friendly and extensible. ComsystanJ combines already known algorithms and newer methods for generalizable analysis of 1D signals, 2D images and 3D volume data including the generation of data sets such as signals and images for testing purposes. It is based on the framework of the open-source image processing software Fiji and ImageJ2. ComsystanJ plugins are macro recordable and are maintained as open-source software. ComsystanJ includes effective surrogate analysis in all dimensions to validate the features calculated by the different algorithms. Future enhancements of the project will include the implementation of parallel computing for image stacks and volumes and the integration of artificial intelligence methods to improve feature recognition and parameter calculation.

## Introduction

Complex systems arise in nature through nonlinear and often repetitive interactions of physical parameters. Recently, the Nobel Prize in Physics 2021 was awarded for research on complex systems with a focus on climate and climate change [1]. Physiological processes, such as phenomena in nature, are complex systems. The rhythm of the human heart, for example, is a consequence of the nonlinear interaction of electrical excitations of a huge number of cardiac muscle cells that are modulated by the autonomic nervous system via a cardiac pacemaker. Therefore, the electrocardiogram itself or beat-to-beat intervals can be successfully investigated by nonlinear 1D signal analysis [2, 3]. Cardiac autonomic neuropathy is an example of a neuropathology that manifests as an arrhythmia and therefore can be identified using linear or nonlinear 1D signal analysis [4, 5]. Other examples from medicine include electroencephalogram or postural sway recordings and their analyses [6–8].

In other scientific fields, such as oncology or geology, dynamical systems cannot be studied directly in the time domain because the time periods are too long for experimental measurements. However, the outcome of such nonlinear dynamical systems can be investigated by their morphological characteristics with 2D images and/or 3D image volumes [9, 10]. Interestingly, 1D morphological analysis is also highly relevant in oncology, mainly because of the full compatibility of 1D analysis with irregularly shaped tumour regions of interest (ROI) in medical images [11]. Another advantage of 1D analysis is its directional sensitivity [11].

A particular example of this former research strategy is at the core of newly developed technologies for assessing neurodivergent and mental health conditions in children (e.g. attention deficit hyperactivity disorder and autism spectrum disorder). Human behaviour and cognition emerge from complex “brain-body-environment” multi-scale interactions. These interactions are controlled by goal-directed and anticipatory mechanisms, known as executive functions (EF). Atypical EF patterns are the most common features associated with neurodivergent and mental health conditions in children. Notably, these atypical EF patterns can be accurately detected using complexity measures such as the fractal dimension and lacunarity [12].

In 2D, the new fractal fragmentation index FFI quantifies the degree of object fragmentation or compaction [13]. At the same time, FFI can identify for each object separately the deviation of its shape from a Euclidean geometric shape. FFI has been successfully applied to study deforestation at the level of counties [13, 14], territorial administrative units [15], or mountain groups [16, 17], showing in all cases that the fractal fragmentation of forests increases as a result of deforestation.

At present, there is no single, comprehensive and continuously maintained software for nonlinear and complexity analysis that can be operated without programming knowledge. The field is quite scattered with proprietary software (e.g. MATLAB, Dataplore) and open-source software or freeware (e.g. TISEAN, PhysioNet, FracLac).

MATLAB https://www.mathworks.com is a programming language with a focus on effective matrix computations. It can be applied to any dimensional input data, but requires scientific programming experience. There are toolboxes and additional libraries for e.g. Detrending fluctuation analysis, Approximate entropy, Sample entropy, Multiscale Entropies, and Lyapunov exponents including surrogates.

Dataplore https://www.ixitos.com/produkte/dataplore is mainly a 1D statistical signal processing software for the end user with a graphical user interface and a macro functionality. It contains many linear statistical methods such as statistical tests, signal manipulation, filtering, wavelet transforms, but only few nonlinear analysis algorithms such as recurrence plots, correlation dimension, Lyapunov exponents and surrogates. The latest version 2.2–3 was released in 2007.

TISEAN https://www.pks.mpg.de/tisean is a dedicated command line software project for 1D time signal analyses of nonlinear deterministic or chaotic systems and includes phase space reconstructions, Lyapunov exponents, correlation dimensions, Rényi entropies and surrogate methods. The latest version 3.0.1 was released in 2007.

PhysioNet https://physionet.org is a research database of complex physiological 1D signals and hosts a number of single software files written in MATLAB, Python or C for nonlinear analyses of physiological time series, especially and mainly for ECG recordings https://physionet.org/about/software.

FracLac https://imagej.nih.gov/ij/plugins/fraclac/FLHelp/Introduction.htm is an end user friendly ImageJ/ImageJ1 plugin for computing fractal dimensions and lacunarities of 2D images with box-counting. Binary and greyscale algorithms are supported. Thanks to a built-in legacy layer for running ImageJ1 plugins, it can still be used in Fiji or ImageJ2. The latest version 2.5 was released in 2012.

Multifrac https://www.ivangtorre.com/software is a new and end user friendly ImageJ2 plugin for computing mono- and multifractal dimensions, including lacunarity and some entropies, in 2D and 3D [18].

In summary, there is no regularly updated software package that contains a large number of algorithms and is easy to use at the same time. ComsystanJ (Complex Systems Analysis for ImageJ) was created to fill this gap. It is platform independent, user friendly for the inexperienced user, macro usable and expandable for the experienced programmer. An exclusive new feature is the support of surrogate analysis for digital images and image volumes. Surrogate analysis is implemented in ComsystanJ without the need to use another ImageJ/Fiji plugin or software. The noise sensitivity can be checked for each algorithm and its specific settings by using a dedicated plugin that adds different types and intensities of artificial noise. Also new is the generalizable support of 1D time signals as well as 2D images and 3D image volumes. It has been developed with individual plugins for each functionality and uses the plugin framework of Fiji/ImageJ2. It is written in Java and hosted as an open-source GitHub project at https://github.com/comsystan/comsystanj.

## Material and methods

### Fiji/ImageJ2 ecosystem

Fiji/ImageJ2 https://imagej.net is the successor of the popular scientific image processing software ImageJ developed by Wayne R. Rasband [19, 20]. ImageJ and Fiji are widely used worldwide and are standards in scientific image processing. There exists a huge user base, a user forum https://forum.image.sc, a large list of plugin extensions https://imagej.net/list-of-extensions and several additional implementations. The current development of Fiji is based on ImageJ2, which has a completely different internal structure than the original ImageJ1. It contains several packages or libraries that can also be used independently of each other if required. A legacy layer ensures that ImageJ1 functionalities are also supported under the new concept of ImageJ2 https://imagej.net/libs/imagej-legacy. The ImageJ ecosystem [21] mainly covers 2D image processing and 3D image stack processing, including 4D applications if time is taken as an additional dimension. Unfortunately, 1D analyses of time signals are not truly an area of interest in this ecosystem, although the new ImageJ2 structure has been developed to be dimension independent. This makes it easy to add 1D processing plugins without having to use or develop another framework. A 1D equivalent ecosystem to Fiji/ImageJ for signal processing does not exist and users have to use proprietary software or individual files with different programming languages suitable only for well-trained scientific programmers.

### ComsystanJ

ComsystanJ shows that 1D signal processing is also effectively possible with the Fiji/ImageJ2 framework. It combines 1D, 2D and 3D nonlinear algorithms to investigate complex systems regardless of dimensionality. One of the advantages of ComsystanJ is that the end user finds common and similar functionalities in all plugins and does not have to switch between and learn different programming languages. Surrogate analysis, which is typically only applied to 1D temporal signals, is supported for all dimensions.

ComsystanJ includes

(i) some common functionalities and linear algorithms such as:

signal-, image-, volume generation, resampling, simple mathematics, filtering, noise addition, FFT, autocorrelation, descriptive statistics, event detection, QRS peak detection, standard HRV measurements, symbolic aggregation,

(ii) common nonlinear and complexity analysis methods for signals and images such as:

Poincare plot, Detrended fluctuation analysis, Hurst coefficient, Katz-, Petrosian-, Sevcik-, Higuchi-, FFT-, Box counting-, Minkowski-, Correlation-, Generalized dimensions, Largest Lyapunov exponent, Recurrence Quantification Analysis, Lacunarity, Succolarity, Approximate-, Sample-, Permutation entropy, Allometric scaling, and Surrogates

and (iii) some very new (partly unpublished) algorithms such as:

Kolmogorov complexity, Logical depth, Generalized entropies (SE, H1, H2, H3, Rényi, Tsallis, SNorm, SEscort, SEta, SKappa, SB, SBeta, SGamma), Fractal fragmentation indices, Pyramid-, RSE-, Directional correlation-, Directional Higuchi-, and Tug of war dimension.

### Surrogate analysis

ComsystanJ includes comprehensive surrogate analyses in 1D, 2D and 3D. Surrogates are generated by modifying the input data using methods such as shuffling or power spectral methods and are used extensively in nonlinear 1D time signal analysis [22]. Shuffling or randomizing the phase before inverse Fourier transformation does not change linear measures such as the mean, median or standard deviation but does have an effect on methods that take correlations into account. This makes it possible to check whether correlations exist within a signal or not. From a mathematical point of view, there is no reason to limit surrogate tests to only one dimension. However, surrogates are not routinely used in 2D image processing. Here, we want to give a plausible explanation for this fact.

1D time signals such as auditory signals are usually visualized as two-dimensional plots and not represented, e.g., as acoustic information for our sense of hearing. Hearing would be one-dimensional in itself, but a graph is not the signal itself; it is a graphic representation suitable for our sense of sight. Therefore, we cannot detect or decide whether correlations are present in such a signal, and our sense of sight can hardly distinguish a surrogate signal from the original signal. Consequently, surrogate analysis is useful because we can use mathematics or algorithms to decide whether correlations exist or not. Most ComsystanJ 1D plugins include surrogate analysis by selecting an additional surrogate method and the number of surrogates in the GUI. Then, the plugin calculates the specific nonlinear parameter, generates the number of surrogates and shows their individual results, their average value and the standard deviation. For the end user, this is a convenient way to achieve maximum results with a single run of a plugin.

2D images of a scene or an object are perfect representations that correspond to the human sense of sight. We can easily see correlations in an image, and surrogates of images (e.g. by randomly shuffling the pixels) can obviously be distinguished. This simple fact seems to be the reason for avoiding surrogates for images, even if the algorithms used for nonlinear analyses do not have sensitivities corresponding to human senses.

From a mathematical point of view, it should not make any difference whether a person has an adequate sense to recognise correlations or not. Thus, ComsystanJ also provides surrogates for analysing 2D images and 3D image stacks. With ComsystanJ, we offer scientists the possibility to use surrogates also for 2D and 3D, as the reliability of nonlinear measures can be tested and further insights can be easily gained.

## Results

### Installation

One of the most important advantages and successes of the ImageJ and Fiji/ImageJ2 ecosystem is that installing a plugin is as easy as copying the plugin file into a plugin subfolder. Thus, it is only necessary to download a ComsystanJ release from the GitHub repository https://github.com/comsystan/comsystanj/releases. Unpack the *.zip file and copy the whole folder to the plugins folder of Fiji. Alternatively, the ComsystanJ *.jar files can be imported using the Fiji command Plugins/Install. Starting Fiji, the ComsystanJ plugins appear under Plugins/ComsystanJ. Fiji should be up to date, and the SCIFIO option (Edit/Options/ImageJ2) must be enabled. To update ComsystanJ, the old ComsystanJ folder must be replaced with a new folder. More than one ComsystanJ folder in Fiji should be avoided.

It is recommended to download a clean copy of the latest Fiji version from the Fiji archive https://downloads.imagej.net/fiji/archive. The SCIFIO option (Edit/Options/ImageJ2) must be enabled in Fiji. Do not use old versions or updated versions of Fiji, as the updater is not always successful.

### Set of plugins

More than 60 plugins are separated into 1D, 2D and 3D plugins. A 3D image stack can be used as input for both 2D and 3D plugins. It might be processed with 2D functionalities slice per slice using 2D plugins or in three dimensions using 3D plugins. A list of the available plugins and functionalities can be found in Tables 1–3. Each set of plugins contains some plugins that are not intended for nonlinear analyses, such as opening, generation and preprocessing. The number of these plugins is very limited for 2D and 3D plugins, as Fiji/ImageJ2 and its functionalities can be used directly. For 1D plugins, this number is higher to cover most of the usually required preprocessing steps for signals. A detailed description of the plugins can be found on the homepage of ComsystanJ https://comsystan.github.io/comsystanj.

**Table 1:** List of 1D plugins and functionalities.

1D plugin	Functionality
Sequence opener	Opens signal/s from a comma delimited text file.
Sequence generator	Constant, Sine, Square, Triangle, Sawtooth, Gaussian and Uniform noise, Discrete chaotical maps (Logistic, Henon, Cubic, Spence), Fractional Gaussian noise with variable Hurst coefficient using Davis and Harte autocorrelation method DHM, Fractional Gaussian motion with variable Hurst coefficient using spectral synthesis method SSM, Weierstraß-Mandelbrot signals with variable fractal dimension, Binary Cantor dusts with variable fractal dimension.
Cut out	Cutting out of a sub-signal.
Resampling	Down- or Up-sampling (Down-sampling is useful for large time signals to reduce computation times).
Mathematics	Differentiation, Integration, Exp, Ln, Log, Sin, Cos, Tan.
Filter	Moving average or Moving median. (Equivalent to low-pass filtering).
Noise	Adding noise: Shot, Salt&Pepper, Uniform, Gaussian, Rayleigh, Exponential. (For validating features and parameters).
Surrogates	Shuffle, Gaussian, Random phase, AAFT. (For validating features and parameters).
FFT	Fast Fourier transform. (Power- or Magnitude spectrum can be displayed).
Autocorrelation	Displays the autocorrelation. (Can be used to find estimates for time delays).
Statistics	Computes descriptive statistics: N, Min, Max, Median, RMS, Mean, SD, Kurtosis, Skewness, Sum, Sum of squares.
Event detection	Detects events such as peaks, valleys, slopes, or QRS peaks, output can be the event time, event value, interval, height, energy, or delta height, threshold or moving average curves MACs, (QRS detection based on the Chen&Chen or OSEA algorithm).
QRS peak detection (from file)	Detects QRS peaks and peak to peak intervals of ECG data, plugin expects ECG files (Holter *.raw files) from disk as input.
Standard HRV measurements	MeanHR, MeanNN, SDNN, SDANN, SDNNI, HRVTI, RMSSD, SDSD, NN50, PNN50, NN20, PNN20, ULF, VLF, LF, HF, LFnorm, HFnorm, LF/HF, TP.
Poincare plot	Poincare plots. (Display of delay plots).
Lyapunov exponent	Largest Lyapunov exponent with Rosenstein’s or Kantz’s algorithm. (Time delay embedding to measure the divergence of initially close phase space trajectories).
Recurrence quantification analysis	Recurrence plot of a time delay embedding and corresponding diagonal measures: RR, DET, RATIO, DIV, Lmean, Lmax, ENT and vertical measures: LAM, TT. (Sensitive to the lengths and numbers of recurrences of a phase space trajectory) [23].
DFA	Detrended fluctuation analysis. (Determines the self-affinity of signals with long-term memory).
Hurst coefficient	SD-power spectrum density, lowPSDwe-PSD using only lower frequencies (low), parabolic windowing (w) and end matching (e) (bridge detrending), SSC—signal summation conversion method for discriminating fGn from fBm signals, Disp—dispersional method, SWV—scaled windowed variance (mean of SD’s), bdSWV—scaled windowed variance (mean of SD’s) with bridge detrending. (A measure for signals with long-term memory, related to the fractal dimension).
Higuchi dimension	Genuine 1D Higuchi fractal dimension. (Measure of self-affinity and space filling properties).
Tug of war dimension	Fractal dimension of binary signal/s. (Computes a set of hash functions and second moments for estimating the fractal dimension).
Katz dimension	Fractal dimension. (Rough estimate of the fractal dimension—fast).
Petrosian dimension	Fractal dimension of binary signal/s. (Rough estimate of the fractal dimension—fast),
Sevcik dimension	Fractal dimension, (Rough estimate of the fractal dimension—fast).
RSE dimension	Roughness scaling extraction fractal dimension. (Variations of data point values at different scales).
Sample entropy	Sample or Approximate entropy. (Closely related to the Kolmogorov entropy and useful for low data point numbers).
Permutation entropy	Permutation entropy, Permutation entropy per symbol, Normalized permutation entropy, Sorting entropy. (Uses the distance between the actual value and previous values).
Generalised entropies	SE, H1, H2, H3, Rényi, Tsallis, SNorm, SEscort, SEta, SKappa, SB, SBeta, SGamma. (Computes a large list of entropies with a selectable range of power exponents).
Kolmogorov complexity	Kolmogorov complexity and Logical depth. (Estimated by lossless ZLIB or GZIB compression).
Allometric scaling	Double log plot of aggregated variances and means.
Symbolic aggregation	Aggregation to a 2D colour image.

**Table 2:** List of 2D plugins and functionalities.

2D plugin	Functionality
Image opener	Single image or image stack, 8bit grey or 24bit RGB colour
Image generator	Random, Gaussian, Sine—radial, Sine—horizontal, Sine—vertical, Constant, Fractal surface—Fourier (FFT) or Midpoint displacement (MPD) or Sum of sine method, Hierarchical random maps, Fractal random shapes, Fractal Iterated function system (IFS)—Menger, Sierpinski, Mandelbrot islands/lakes, Koch snowflake, Fern, Heighway dragon.
Filter	Gaussian blur, Mean, Median, FFT-Lowpass. (For validating features and parameters).
Noise	Adding noise: Shot, Salt&Pepper, Uniform, Gaussian, Rayleigh, Exponential. (For validating features and parameters).
Surrogates	Shuffle, Gaussian, Random phase, AAFT. (For validating features and parameters).
Box counting dimension	Fractal dimension with box counting. (Standard algorithm for estimating the fractal dimension of digital images. It is a measure of self-affinity and space filling properties).
Pyramid dimension	Fractal dimension with image pyramids. (Closely related to the box counting algorithm).
Minkowski dimension	Fractal dimension, dilation, blanket or variation method. (Uses morphological dilation to create different scales).
Correlation dimension	Fractal dimension, grey value mass algorithm, raster box scanning or -sliding box scanning. (Classical pair wise occurrence counting at different distances).
Directional correlation dimension	Directional dependent fractal correlation dimension. (A new method for determining the fractal correlation dimension in different directions).
Generalized dimensions	Generalized fractal dimensions, binary or grey value mass algorithm, raster or sliding box scanning. (Computes several fractal dimensions with a selectable range of power exponents–closely related to the Correlation dimension).
Higuchi dimension 1D	Fractal dimension using extracted 1D grey value profiles [24]. (Uses the genuine 1D algorithm).
Higuchi dimension 2D	Fractal dimension with 2D algorithms, k-fold differences, multiplicated differences, squared differences, direct differences [25]. (2D generalisations of the genuine 1D algorithm).
FFT dimension	Fractal dimension, circular average of k values, mean of separate line scans, integrated line scans. (Uses the decay of the power spectrum to estimate the fractal dimension).
Tug of war dimension	Fractal dimension using coordinates of data points [26]. (Computes a set of hash functions and second moments for estimating the fractal dimension).
Lacunarity	Raster/sliding box scanning or Tug of war method. (A measure that is sensitive to the number and size of gaps in an object).
Succolarity	Raster box or sliding box scanning. (A measure of how an object can be virtually flooded with water from different sides).
Fractal fragmentation indices	Fractal fragmentation index FFI, Fractal fragmentation and disorder index FFDI, Fractal tentacularity index FTI. (New fractal algorithms for measuring the fragmentation and tentacularity of objects at different scales).
Generalised entropies	SE, H1, H2, H3, Rényi, Tsallis, SNorm, SEscort, SEta, SKappa, SB, SBeta, SGamma. (Computes a large list of entropies with a selectable range of power exponents).
Kolmogorov complexity	Kolmogorov complexity and Logical depth. (Estimated by lossless ZIP, ZLIB, GZIB or LZW, PNG compression).

**Table 3:** List of 3D plugins and functionalities.

3D plugin	Functionality
3D Image volume opener	Image stack, 8bit grey or 24bit RGB colour
3D Image volume generator	Random, Gaussian, Constant, Fractal surface—Fourier (FFT) or Midpoint displacement (MPD), Fractal iterated function system (IFS)—Menger cube, Sierpinski pyramid or Mandelbrot cube.
3D Filter	Gaussian blur, Mean, Median, FFT-Lowpass. (For validating features and parameters).
3D Noise	Adding noise: Shot, Salt&Pepper, Uniform, Gaussian, Rayleigh, Exponential. (For validating features and parameters)
3D Surrogates	Shuffle, Gaussian, Random phase, AAFT. (For validating features and parameters).
3D Box counting dimension	Fractal dimension with box counting. (Standard algorithm for estimating the fractal dimension of digital image volumes).
3D Minkowski dimension	Fractal dimension, dilation, blanket or variation method. (Uses morphological dilation to create different scales).
3D Correlation dimension	Fractal dimension, grey value mass algorithm, raster box scanning or sliding box scanning. (Classical pair wise occurrence counting at different distances).
3D Generalized dimensions	Generalized fractal dimensions, binary or grey value mass algorithm, raster or sliding box scanning. (Computes several fractal dimensions with a selectable range of power exponents–closely related to the Correlation dimension).
3D Higuchi dimension	Fractal dimension with 3D algorithms, k-fold differences, multiplicated differences, squared differences, direct differences. (Generalisations of the genuine 1D algorithm).
3D FFT dimension	Fractal dimension with 3D FFT. (Uses the decay of the power spectrum to estimate the fractal dimension).
3D Tug of war dimension	Fractal dimension with 3D Tug of war algorithm [26]. (Computes a set of hash functions and second moments for estimating the fractal dimension).
3D Lacunarity	Raster/sliding box scanning or Tug of war method. (A measure that is sensitive to the number and size of holes in an object).
3D Fractal fragmentation indices	Fractal fragmentation index FFI, Fractal fragmentation and disorder index FFDI, Fractal tentacularity index FTI. (New fractal algorithms for measuring the fragmentation and tentacularity of objects at different scales).
3D Generalised entropies	SE, H1, H2, H3, Rényi, Tsallis, SNorm, SEscort, SEta, SKappa, SB, SBeta, SGamma. (Computes a large list of entropies with a selectable range of power exponents).
3D Kolmogorov complexity	Kolmogorov complexity and Logical depth. (Estimated by lossless ZIP, ZLIB, GZIB or LZW compression).

### Description of 1D signal plugins

The internal representation of a signal for ComsystanJ and Fiji/ImageJ2 is a table (SciJava class DefaultGenericTable) and especially a table column. The first row entry represents the column header and can simply be the name of the signal or file. Several signals correspond to distinct columns. However, the first column is reserved for the corresponding row headers, which in most cases are ascending integer numbers representing the sample number.

Data files for signals must meet some requirements resulting from the internal structures of Fiji/ImageJ2. Firstly, the signal files must be comma separated text files. The first row and the first column can contain any characters, the data values must be real numbers with a point as decimal place. Secondly, each column must have the same size. If the number of data values in a column is shorter, the missing data values must be filled with the character NaN.

Files that meet these requirements must be opened with the ComsystanJ Opener plugin and are displayed as a table. This table contains the signal data and is the source for the signal plugins used in the following. A graphical display of the signal(s) can optionally be shown, but is not the source for the signal plugins and can be omitted for very large data series if memory is limited.

A large number of artificially generated signal types can be generated with the signal generator plugin to have a convenient possibility for comparisons. Signal types might be linear (Constant, Sine,…), random (Gaussian or uniform), chaotic (Logistic, Henon…), fractal (fGn, fGm….) or binary (Cantor). A list of 1D functionalities can be seen in Table 1.

The GUI of a typical analysis plugin consists of parameter settings for the specific algorithm, followed by analysis options, display options and process options common to all 1D plugins.

Analysis options include the choice of simply applying the algorithm to the entire signal or to subsequent or sliding boxes (with adjustable box length), resulting in a list of result values for each window. The latter two options are a convenient way to examine the development of the result values over time without having to write a macro for it. Surrogates (with adjustable number of surrogates) can be activated for the entire signal range and then the analysis is additionally carried out automatically for several surrogates and individual surrogate result values as well as an average surrogate value are included in the result table without having to write a macro for it.

The display options decide whether algorithm dependent graphs or windows are displayed or not and whether already displayed windows are overwritten or not.

The process options include the possibility to activate an immediate preview when any GUI element is changed by the user. Pressing the OK button finally processes all available signals without having to write a macro for that.

**Fig 1:**
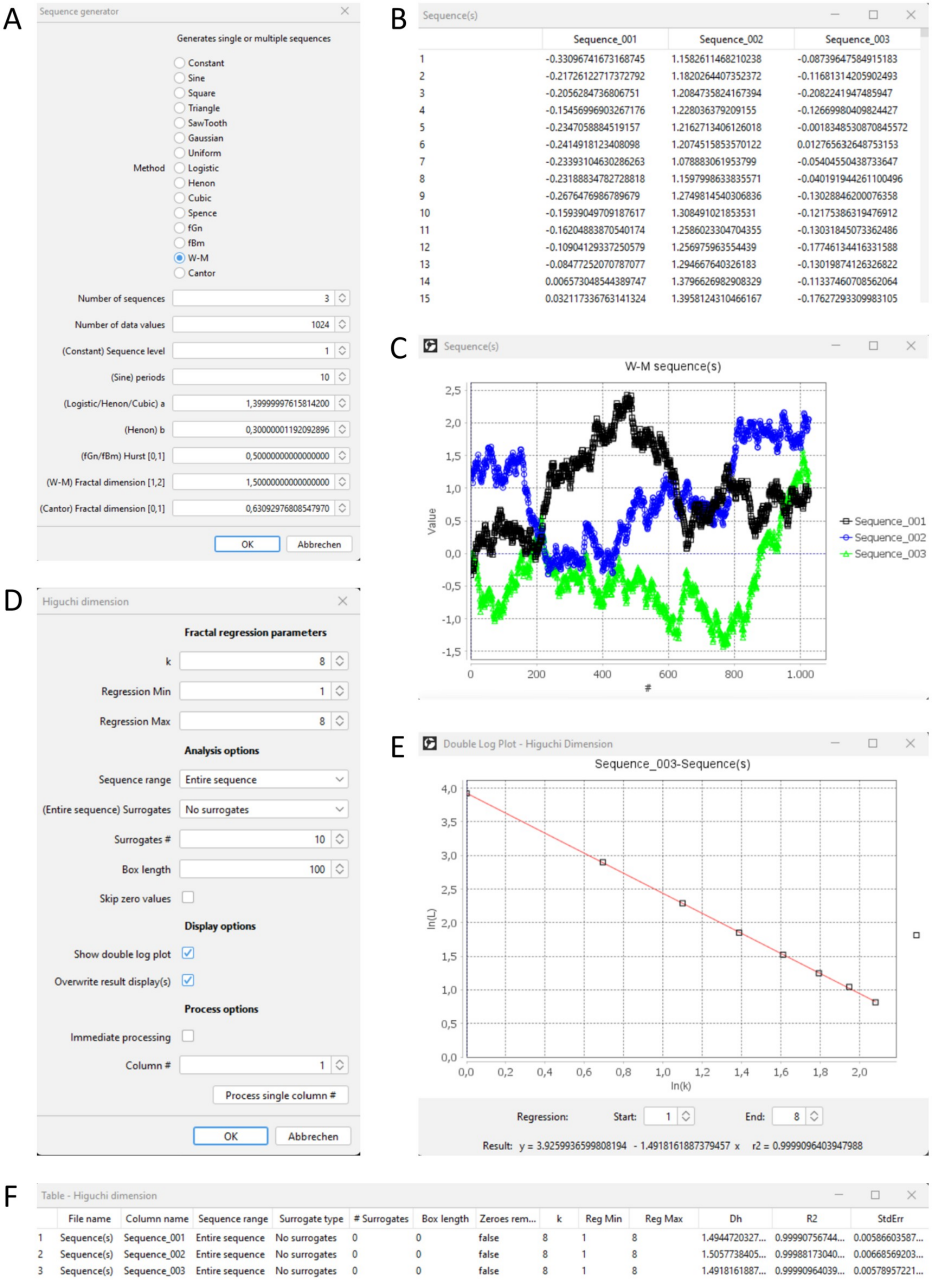
Example of 1D signal analysis. (A) GUI for generating 1D sequences. (B) Table of generated sequence data values. (C) Graphical representation of sequences with a predefined theoretical fractal dimension of 1.5. (D) GUI for determining the Higuchi dimensions. (E) Double logarithmic plot of summed up sequence lengths and delay parameter k. (F) Result table including the Higuchi dimensions Dh (around 1.5) for all three sequences.

Typical GUIs of a signal plugin can be seen in Fig 1A and 1D.

### Example of 1D signal analysis

Close all open Windows or restart Fiji and generate a set of signals with the following command and parameter settings:

Plugins/ComsystanJ/1D Sequence(s)/Sequence generator (see Fig 1A), Method: W-M (Weierstrass-Mandelbrot function), Number of sequences: 3, Number of data values: 1024, (W-M) Fractal dimension [1, 2]: 1.5

The remaining options can be left as defaults. Press the OK button and confirm the Display option with Yes. A set of signals will be shown in a table (Fig 1B) and additionally a window with graphs will be displayed (Fig 1C). Please note that actual values and graphs vary with each generation.

The subsequent determination of e.g. the Higuchi dimension can be done with:

Plugins/ComsystanJ/1D Sequence(s)/Higuchi dimension (see Fig 1D), k: 8, Regression Min: 1, Regression Max: 8, Signal range: Entire sequence, (Entire signal) Surrogates: No surrogates, Surrogates #: 10, Box length: 100, Skip zero values: Disabled, Show double log plot: Enabled, Overwrite result display(s): Enabled, Immediate processing: Disabled, Column #: 1

A preview can be started by pressing the button Process single column #. A result table and a double log plot (Fig 1E) will be displayed. The Column # may be changed and again a preview can be started. Finally, by pressing the OK button, all three signals will be processed and the results will be displayed (Fig 1F). The values in the Dh column represent the determined fractal dimensions and should be about 1.5. The column R2 shows the values of the coefficient of determination of the linear regressions.

### Description of 2D image plugins

2D images can be opened either with the genuine Fiji/ImageJ2 commands or with the ComsystanJ image opener plugin. The latter has the advantage that multiple images can be selected individually and when multiple images are selected, they are automatically opened as an image stack. Internally, an image or image stack is held in the primary ImageJ2 dataset container called Dataset, which provides access to pixels and metadata. The SCIFIO option must be activated in Fiji to ensure the ImageJ2 image format for input and output.

An image generator plugin with more than 20 different image types is available. It includes linear types (Constant, Sine,…), random (Gaussian or uniform), fractal surfaces (FFT or MPD) or binary IFS types (Menger, Sierpinski,…..). Images can be created with selectable size, colour type and number of images and can be used for comparison purposes.

A filter plugin and a noise plugin are provided, as filtering and adding noise are two direct and commonly used methods for testing nonlinear and complex analysis algorithms. Other methods can be used from the Fiji/ImageJ2 ecosystem.

A list of 2D functionalities can be seen in Table 2.

The typical GUI for image plugins consists of method specific settings such as the regression range in the double log plot or binary/grey value analysis options, followed by display options and process options available in each GUI. The display options can be used to set whether method-specific windows such as the double log plot or result images are displayed and whether previously displayed windows are overwritten or not. Process options include an instant preview option where calculations are started as soon as a GUI element is changed by the user, and the ability to start a preview calculation with a selectable image number (for image stacks). By pressing the OK button, all available images are processed automatically without having to write a macro.

**Fig 2:**
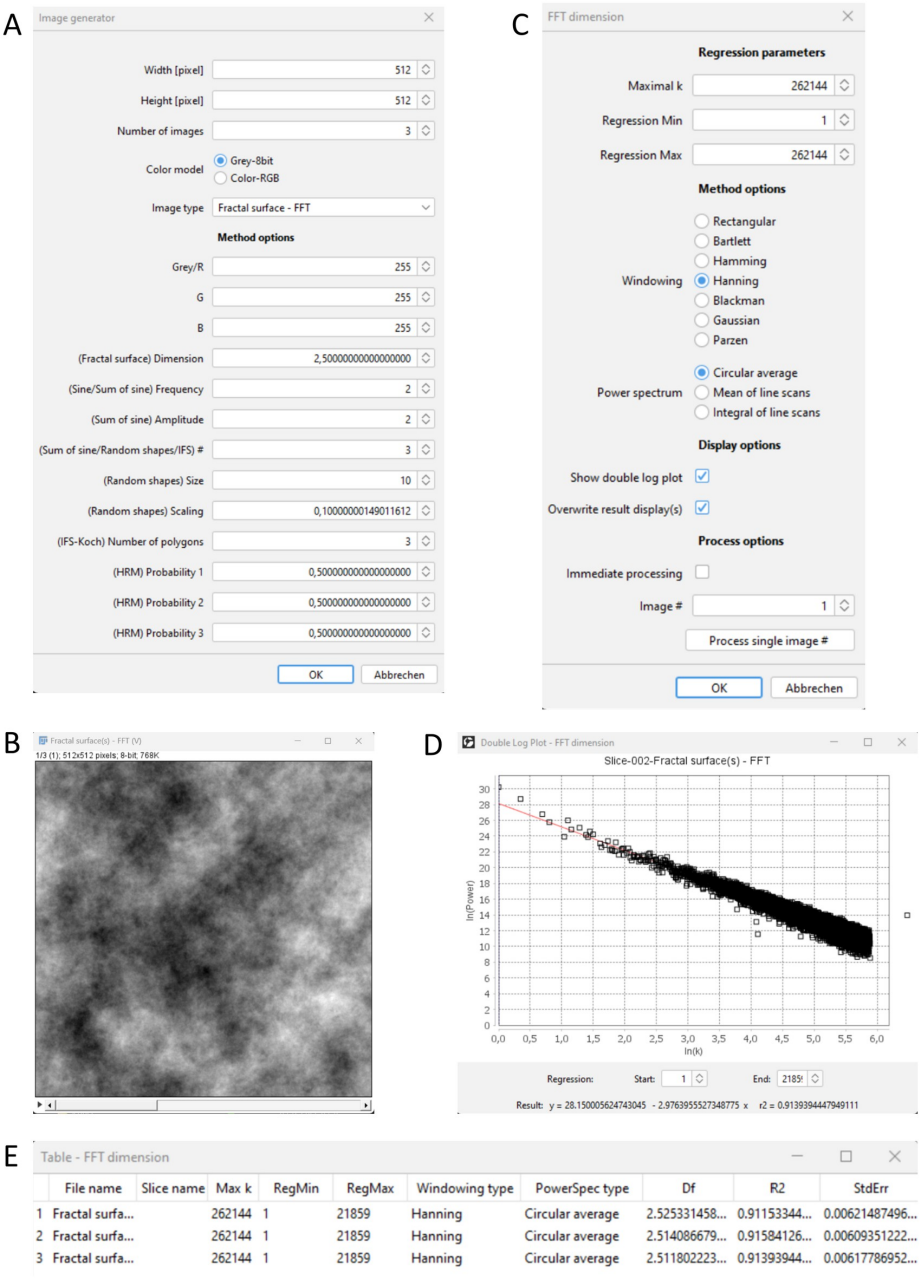
Example of 2D image analysis. (A) GUI for generating 2D images. (B) Generated grey value images with a predefined theoretical fractal dimension of 2.5. (C) GUI for determining the FFT dimensions. (D) Double logarithmic plot of summed up spectral power values and frequency parameter k. (E) Result table including the FFT dimensions Df (around 2.5) for all three images.

Example GUIs of an image plugin can be seen in Fig 2A and 2C.

### Example of 2D image analysis

Close all open windows or restart Fiji and generate a set of images with the following command and parameter settings:

Plugins/ComsystanJ/2D Image(s)/Image generator (see Fig 2A), Width [pixel]: 512, Height [pixel]: 512, Number of images: 3, Color model: Grey-8bit, Image type: Fractal surface–FFT, Grey/R: 255, (Fractal surface) Dimension: 2.5

The remaining options can be left as default values. Press the OK button and an image stack will be displayed (Fig 2B). Each image has an individual pattern but the theoretical fractal dimension is always 2.5. Actual images vary with each generation.

A subsequent analysis could be the determination of the fractal dimension using FFT (fast Fourier transform) with:

Plugins/ComsystanJ/2D Image(s)/FFT dimension (see Fig 2C), Maximal k: 262144, Regression Min: 1, Regression Max: 262144, Windowing: Hanning, Power spectrum: Circular average, Show double log plot: Enabled, Overwrite result display(s): Enabled, Immediate processing: Disabled, Image #: 1

A preview can be started by pressing the button Process single image #. A result table and a double log plot (Fig 2D) will be displayed. The Image # may be changed and again a preview can be started. Finally, by pressing the OK button, all three images will be processed and the results will be displayed (Fig 2E). The values in the Df column are the determined fractal dimensions and should be about 2.5. The column R2 shows the values of the coefficient of determination of the linear regressions.

### Description of 3D image volume plugins

Equivalently to 2D, a 3D image stack or volume can be opened either with the genuine Fiji/ImageJ2 commands or with the corresponding ComsystanJ volume opener plugin. The SCIFIO option must be activated in Fiji.

A 3D volume generator plugin is available to generate fractal 3D grey value volumes, a Menger cube, multiple Sierpinski pyramids and a variation of 3D Mandelbrot cubes.

A comprehensive list of 12 plugins for 3D nonlinear and complexity analysis are included. These 3D plugins are very suitable for determining fractal dimensions or other complexity measures of grey scale image volumes and can be directly used for cranial MRIs, for example.

Thus, these algorithms appear in ComsystanJ in 1D, 2D as well as in 3D, representing a suitable set of algorithms for any dimensionality. A list of 3D functionalities can be seen in Table 3.

**Fig 3:**
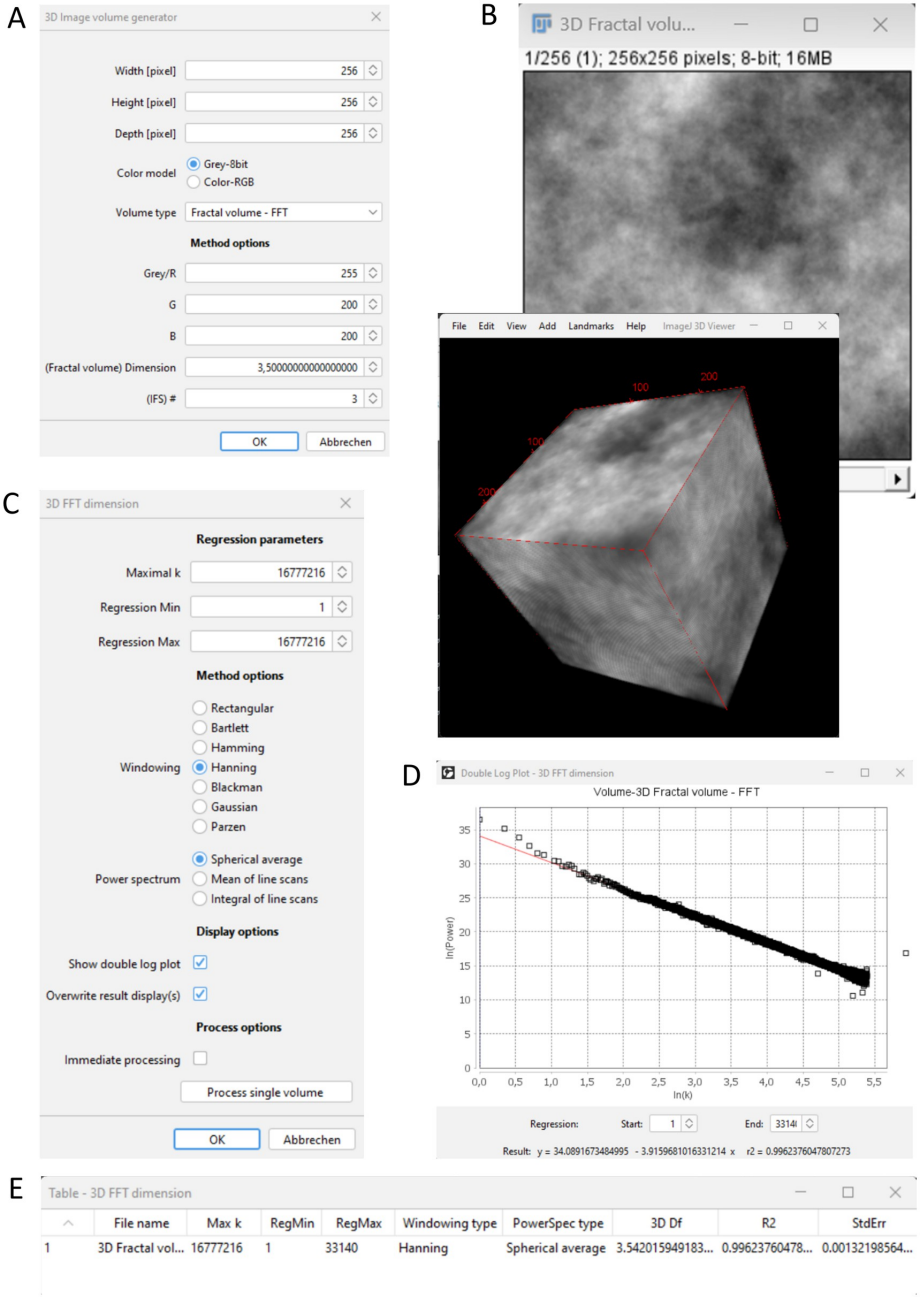
Example of 3D image volume analysis. (A) GUI for generating a 3D image volume. (B) Generated grey value image volume with a predefined theoretical fractal dimension of 3.5 and a three-dimensional representation. (C) GUI for determining the 3D FFT dimension. (D) Double logarithmic plot of summed up spectral power values and frequency parameter k. (E) Result table including the 3D FFT dimension 3D Df (around 3.5) for the image volume.

Example GUIs can be seen in Fig 3A and 3C.

### Example of 3D image volume analysis

Close all open windows or restart Fiji and generate an image stack volume with the following command and parameter settings:

Plugins/ComsystanJ/3D Volume/3D Image volume generator (see Fig 3A), Width [pixel]: 256, Height [pixel]: 256, Depth [pixel]: 256, Color model: Grey-8bit, Volume type: Fractal volume–FFT, Grey/R: 255, (Fractal volume) Dimension: 3.5

Please note that increased size settings will increase memory demand. Contrary to the 2D example, the fractal grey value pattern is also smooth along the third depth axis. Optionally, the volume (Fig 3B) can additionally be displayed with Fiji’s Volume Viewer (Plugins/Volume Viewer) or 3D Viewer (Plugins/3D Viewer) (Fig 3B). Actual volumes vary with each generation.

The determination of the fractal dimension using FFT in 3D can be started with:

Plugins/ComsystanJ/3D Volume/3D FFT dimension (see Fig 3C), Maximal k: 16777216, Regression Min: 1, Regression Max: 16777216, Windowing: Hanning, Power spectrum: Spherical average, Show double log plot: Enabled, Overwrite result display(s): Enabled, Immediate processing: Disabled

A preview can be started by pressing the button Process single volume. A results table and a double log plot (Fig 3D) will be displayed. The settings may be changed and a preview can be started again. By clicking the OK button, the entire image volume will be processed in 3D and the results will be displayed (Fig 3E). The value in the 3D Df column should be about 3.5 in this case. The column R2 shows the coefficient of determination of the linear regression.

### Computational expense

The plugins were coded in the most effective way to keep the computational overhead to a minimum. In addition, Java itself is very efficient for processing loops, which are used extensively when scrolling through long time series data or through the two-dimensional grid of a digital image. Signal and image data variables were implemented as generic and effective array classes, costly classes such as the Vector class were avoided. Exemplarily, the following computing times (Table 4) were determined for a standard PC and a high-end workstation including the display of corresponding windows.

**Table 4:** Computational expense. Mean values over three measurements. Standard PC: Windows 11Pro, 64bit, CPU Intel i5-10500 3.10GHz, 6 cores, Graphics Intel UHD 630, RAM 16GB, HD 512GB SSD. Workstation: Ubuntu 22.04.2 LTS, 64bit, CPU AMD Ryzen Threadripper pro 5955wx 4GHz, 16 cores, Graphics GeForce GT1030 2GHD4 LP OC 2GB DDR4, RAM 512GB DDR4-3200, HD 2TB M.2 SSD.

Task	Computational expense	
**1D signals**	**PC**	**Workstation**
Generation of 10 fractal signals with 1024 data points	44.3s	17.1s
Computation of Higuchi dimension	836μs	393μs
Computation of FFT dimension	148μs	109μs
Computation of Kolmogorov complexity	129μs	63μs
**2D image**	**PC**	**Workstation**
Generation of a fractal grey value image with 1024x1024pixel	816μs	592μs
Computation of 2D Higuchi dimension	5.3s	3.4s
Computation of FFT dimension	985μs	639μs
Computation of Kolmogorov complexity	226μs	109μs
**2D image stack/3D volume**	**PC**	**Workstation**
Generation of a fractal grey value image stack with 512x512x512	39.9s	24.1s
Computation of 3D Higuchi dimension	18’43.8s	13’2.1s
Computation of 3D FFT dimension	35.5s	23.7s
Computation of 3D Kolmogorov complexity	10.3s	9.2s

### Description of limitations

The graphical GUIs of the plugins extend the SciJava class ContextCommand and provide an OK and a Cancel button. This is a very convenient and short way to define @parameters like services (UIService, LogService, StatusService, OpService,…) and GUI elements.

The first limitation is that GUI elements and their functionalities cannot be greyed out or switched on and off. For example, the signal and image generator plugins consist of many parameters, but only some parameters are used for a specific type of signal or image. The end user could easily be confused by the long list of parameters and may not be able to intuitively find the right parameter settings. To reduce this disadvantage, we named parameters in the ComsystanJ plugins with a leading expression in round brackets, indicating to which type of setting this individual parameter belongs. This is of course unsatisfactory, but might be solved by future versions of the class ContextCommand.

A second limitation of the ContextCommand is that it is a modal UI window that does not offer the possibility to conveniently preview the plugin functionalities. It is not possible, for example, to change the linear range of the regression directly in the double log plot in a preview mode and then press OK for the final computations. These settings can only be changed after the plugin has been executed and the GUI and the plugin may have to be restarted. The SciJava class InteractiveCommand does not have this restriction because it is not modal, but unfortunately plugins can no longer be used in macros. Therefore, Fiji/ImageJ2 plugins have either good preview functionality or macro functionality and cannot be constructed to do both. To ensure macro functionality, the ComsystanJ plugins unfortunately lack some preview convenience.

The third known limitation is a display problem of Fiji/ImageJ2. Colour images are sometimes incorrectly displayed with a red cast, and grey images are sometimes displayed with oversaturated grey values. This issue might be fixed in future versions of Fiji/ImageJ2.

## Conclusions

ComsystanJ is a set of Fiji/ImageJ2 plugins for the study of nonlinear and complex systems in 1D, 2D and 3D. It offers a convenient user experience for the end user, is open access and is extensible for the experienced user or programmer. More than 60 plugins offer more than 100 parameters and results that can be calculated for the characterisation of the signal, image or image volume under investigation. The surrogate analysis is implemented by default and is also strongly recommended for images and image volumes. ComsystanJ includes common algorithms such as the Box counting dimension and the FFT dimension but also recently developed methods such as the 2D/3D Higuchi dimension, the 1D/2D RSE fractal dimension, and the Kolmogorov complexity.

Here we have given an overview of ComsystanJ, the technical aspects related to its functions, surrogate analysis, GUI design and interaction with the ImageJ2/Fiji ecosystem. Follow-up studies will be more specific and focus on a particular dimensionality, including the presentation of real-world data investigations. In particular, in-depth 1D signal analyses of e.g. ECG, EEG and long duration geological time signals will demonstrate the usefulness of this package for time signals, with the intention of reaching more scientists outside the ImageJ/Fiji imaging community.

The project will be continuously maintained and improved by increasing the number of plugins. In particular, the number of direction-sensitive and ROI-restricted 2D/3D methods will be increased. Currently, ComsystanJ has a small bias towards cardiac or human signal and image analysis, although most features and parameters could be used universally. However, future ComsystanJ enhancements will include generally usable metrics such as the Ruler dimension, Perimeter-area dimension, Mass dimension, Gravelius compactness coefficient, order number methods (Gravelius, Horton, Strahler, Schreve, Scheidegger, Horsfield-Cumming, Graf), and new indices such as a Dendritic complexity index, Fractal allometry index, Fractal connectivity dimension, Lacunarity dispersions index and Internal succolarity. Other future improvements will be parallel computing with special threads for large image stacks and the integration of new machine learning algorithms such as convolutional neural networks, which are already being used successfully for complexity analyses [27–30]. Explainability and interpretability are important concepts in AI-based image and bio-signal analysis. ComsystanJ has the potential to incorporate explainable AI for model performance as part of classification tasks.
